# Supplementary material for: Label tree semantic losses for rich multi-class medical image segmentation
Source: Front Artif Intell. 2026 Jun 16;9:1841639. doi: 10.3389/frai.2026.1841639 (PMC13315184; doi:10.3389/frai.2026.1841639)
Supplement: Supplementary file 1 [file Supplementary_file_1.pdf]

Supplementary Material
Label tree semantic losses for rich multi-class medical image segmentation

Junwen Wang, Oscar MacCormac, William Rochford, Aaron Kujawa, Jonathan Shapey and Tom Vercauteren

Supplementary Table

Table S1: Paired-sample WBP statistical tests on matched test subjects. Δ: proposed method minus comparator. Raw p-values come from two-sided paired t-tests; adjusted p-values use Holm-Bonferroni correction across all predefined WBP headline comparisons. The Raw p and Holm columns report significance markers rather than numeric p-values. Significance markers use raw p-values in the Raw p column and Holm-adjusted p-values in the Holm column: * $p < 0.05$; ns, no significance.

Comparison	Train	Test	Metric	n	Mean Δ	Median Δ	Raw p	Holm sig.
$\mathcal{L}_{wass+seg}$ vs $\mathcal{L}_{seg}$	MB59	MB42	Dice	42	0.0020	0.0021	*	*
$\mathcal{L}_{wass+seg}$ vs $\mathcal{L}_{seg}$	MB59	MB42	NSD	42	0.0055	0.0066	*	*
$\mathcal{L}_{wass+seg}$ vs $\mathcal{L}_{seg}$	MB59	MB42	Dice_small	42	0.0132	0.0096	*	*
$\mathcal{L}_{wass+seg}$ vs $\mathcal{L}_{seg}$	MB59	MB42	NSD_small	42	0.0563	0.0869	*	*
$\mathcal{L}_{wass+seg}$ vs $\mathcal{L}_{seg}$	MB59	AOMIC	Dice	46	0.0035	0.0029	*	*
$\mathcal{L}_{wass+seg}$ vs $\mathcal{L}_{seg}$	MB59	AOMIC	NSD	46	0.0065	0.0087	*	*
$\mathcal{L}_{wass+seg}$ vs $\mathcal{L}_{seg}$	MB59	AOMIC	Dice_small	46	0.0283	0.0298	*	*
$\mathcal{L}_{wass+seg}$ vs $\mathcal{L}_{seg}$	MB59	AOMIC	NSD_small	46	0.0670	0.0975	*	*
$\mathcal{L}_{wass+seg}$ vs $\mathcal{L}_{seg}$	MB59	IXI	Dice	117	0.0032	0.0032	*	*
$\mathcal{L}_{wass+seg}$ vs $\mathcal{L}_{seg}$	MB59	IXI	NSD	117	0.0071	0.0082	*	*
$\mathcal{L}_{wass+seg}$ vs $\mathcal{L}_{seg}$	MB59	IXI	Dice_small	117	0.0181	0.0150	*	*
$\mathcal{L}_{wass+seg}$ vs $\mathcal{L}_{seg}$	MB59	IXI	NSD_small	117	0.0721	0.0942	*	*
$\mathcal{L}_{wass+seg}$ vs $\mathcal{L}_{seg}$	AOMIC	MB42	Dice	42	-0.0002	-0.0004	ns	ns
$\mathcal{L}_{wass+seg}$ vs $\mathcal{L}_{seg}$	AOMIC	MB42	NSD	42	0.0045	0.0057	*	*
$\mathcal{L}_{wass+seg}$ vs $\mathcal{L}_{seg}$	AOMIC	MB42	Dice_small	42	0.0150	0.0043	*	*
$\mathcal{L}_{wass+seg}$ vs $\mathcal{L}_{seg}$	AOMIC	MB42	NSD_small	42	0.0707	0.0905	*	*
$\mathcal{L}_{wass+seg}$ vs $\mathcal{L}_{seg}$	AOMIC	AOMIC	Dice	46	0.0063	0.0064	*	*
$\mathcal{L}_{wass+seg}$ vs $\mathcal{L}_{seg}$	AOMIC	AOMIC	NSD	46	0.0127	0.0151	*	*
$\mathcal{L}_{wass+seg}$ vs $\mathcal{L}_{seg}$	AOMIC	AOMIC	Dice_small	46	0.0705	0.0784	*	*
$\mathcal{L}_{wass+seg}$ vs $\mathcal{L}_{seg}$	AOMIC	AOMIC	NSD_small	46	0.1390	0.1693	*	*
$\mathcal{L}_{wass+seg}$ vs $\mathcal{L}_{seg}$	AOMIC	IXI	Dice	117	0.0004	0.0013	ns	ns
$\mathcal{L}_{wass+seg}$ vs $\mathcal{L}_{seg}$	AOMIC	IXI	NSD	117	0.0096	0.0116	*	*
$\mathcal{L}_{wass+seg}$ vs $\mathcal{L}_{seg}$	AOMIC	IXI	Dice_small	117	0.0600	0.0586	*	*
$\mathcal{L}_{wass+seg}$ vs $\mathcal{L}_{seg}$	AOMIC	IXI	NSD_small	117	0.1355	0.1514	*	*
$\mathcal{L}_{wass+seg}$ vs $\mathcal{L}_{seg}$	IXI	MB42	Dice	42	0.0030	0.0026	*	*
$\mathcal{L}_{wass+seg}$ vs $\mathcal{L}_{seg}$	IXI	MB42	NSD	42	0.0054	0.0072	*	*
$\mathcal{L}_{wass+seg}$ vs $\mathcal{L}_{seg}$	IXI	MB42	Dice_small	42	0.0148	0.0054	*	*
$\mathcal{L}_{wass+seg}$ vs $\mathcal{L}_{seg}$	IXI	MB42	NSD_small	42	0.0514	0.0801	*	*
$\mathcal{L}_{wass+seg}$ vs $\mathcal{L}_{seg}$	IXI	AOMIC	Dice	46	0.0015	0.0016	*	ns
$\mathcal{L}_{wass+seg}$ vs $\mathcal{L}_{seg}$	IXI	AOMIC	NSD	46	0.0055	0.0079	*	*
$\mathcal{L}_{wass+seg}$ vs $\mathcal{L}_{seg}$	IXI	AOMIC	Dice_small	46	0.0353	0.0357	*	*
$\mathcal{L}_{wass+seg}$ vs $\mathcal{L}_{seg}$	IXI	AOMIC	NSD_small	46	0.0643	0.0957	*	*
$\mathcal{L}_{wass+seg}$ vs $\mathcal{L}_{seg}$	IXI	IXI	Dice	117	0.0037	0.0046	*	*
$\mathcal{L}_{wass+seg}$ vs $\mathcal{L}_{seg}$	IXI	IXI	NSD	117	0.0065	0.0087	*	*
$\mathcal{L}_{wass+seg}$ vs $\mathcal{L}_{seg}$	IXI	IXI	Dice_small	117	0.0454	0.0547	*	*
$\mathcal{L}_{wass+seg}$ vs $\mathcal{L}_{seg}$	IXI	IXI	NSD_small	117	0.0726	0.0969	*	*
$\mathcal{L}_{twce+seg}$ vs $\mathcal{L}_{seg}$	MB59	MB42	Dice	42	-0.0020	-0.0014	*	*
$\mathcal{L}_{twce+seg}$ vs $\mathcal{L}_{seg}$	MB59	MB42	NSD	42	-0.0025	-0.0025	*	*
$\mathcal{L}_{twce+seg}$ vs $\mathcal{L}_{seg}$	MB59	MB42	Dice_small	42	-0.0003	0.0014	ns	ns
$\mathcal{L}_{twce+seg}$ vs $\mathcal{L}_{seg}$	MB59	MB42	NSD_small	42	-0.0008	-0.0028	ns	ns
$\mathcal{L}_{twce+seg}$ vs $\mathcal{L}_{seg}$	MB59	AOMIC	Dice	46	0.0019	0.0018	*	*
$\mathcal{L}_{twce+seg}$ vs $\mathcal{L}_{seg}$	MB59	AOMIC	NSD	46	0.0003	0.0004	ns	ns
$\mathcal{L}_{twce+seg}$ vs $\mathcal{L}_{seg}$	MB59	AOMIC	Dice_small	46	0.0007	0.0006	ns	ns
$\mathcal{L}_{twce+seg}$ vs $\mathcal{L}_{seg}$	MB59	AOMIC	NSD_small	46	0.0022	0.0008	ns	ns
$\mathcal{L}_{twce+seg}$ vs $\mathcal{L}_{seg}$	MB59	IXI	Dice	117	0.0014	0.0016	*	*
$\mathcal{L}_{twce+seg}$ vs $\mathcal{L}_{seg}$	MB59	IXI	NSD	117	-0.0006	-0.0005	*	ns
$\mathcal{L}_{twce+seg}$ vs $\mathcal{L}_{seg}$	MB59	IXI	Dice_small	117	-0.0012	-0.0005	ns	ns
$\mathcal{L}_{twce+seg}$ vs $\mathcal{L}_{seg}$	MB59	IXI	NSD_small	117	-0.0005	0.0010	ns	ns
$\mathcal{L}_{twce+seg}$ vs $\mathcal{L}_{seg}$	AOMIC	MB42	Dice	42	-0.0011	-0.0009	*	ns

Comparison	Train	Test	Metric	n	Mean Δ	Median Δ	Raw p	Holm sig.
$\mathcal{L}_{twce+seg}$ vs $\mathcal{L}_{seg}$	AOMIC	MB42	NSD	42	-0.0000	-0.0003	ns	ns
$\mathcal{L}_{twce+seg}$ vs $\mathcal{L}_{seg}$	AOMIC	MB42	Dice_small	42	-0.0025	-0.0029	ns	ns
$\mathcal{L}_{twce+seg}$ vs $\mathcal{L}_{seg}$	AOMIC	MB42	NSD_small	42	0.0126	0.0067	*	ns
$\mathcal{L}_{twce+seg}$ vs $\mathcal{L}_{seg}$	AOMIC	AOMIC	Dice	46	0.0013	0.0014	*	ns
$\mathcal{L}_{twce+seg}$ vs $\mathcal{L}_{seg}$	AOMIC	AOMIC	NSD	46	0.0054	0.0052	*	*
$\mathcal{L}_{twce+seg}$ vs $\mathcal{L}_{seg}$	AOMIC	AOMIC	Dice_small	46	0.0270	0.0289	*	*
$\mathcal{L}_{twce+seg}$ vs $\mathcal{L}_{seg}$	AOMIC	AOMIC	NSD_small	46	0.0678	0.0656	*	*
$\mathcal{L}_{twce+seg}$ vs $\mathcal{L}_{seg}$	AOMIC	IXI	Dice	117	-0.0014	0.0001	ns	ns
$\mathcal{L}_{twce+seg}$ vs $\mathcal{L}_{seg}$	AOMIC	IXI	NSD	117	0.0025	0.0048	*	*
$\mathcal{L}_{twce+seg}$ vs $\mathcal{L}_{seg}$	AOMIC	IXI	Dice_small	117	0.0309	0.0333	*	*
$\mathcal{L}_{twce+seg}$ vs $\mathcal{L}_{seg}$	AOMIC	IXI	NSD_small	117	0.0694	0.0756	*	*
$\mathcal{L}_{twce+seg}$ vs $\mathcal{L}_{seg}$	IXI	MB42	Dice	42	0.0005	-0.0001	ns	ns
$\mathcal{L}_{twce+seg}$ vs $\mathcal{L}_{seg}$	IXI	MB42	NSD	42	-0.0018	-0.0014	*	*
$\mathcal{L}_{twce+seg}$ vs $\mathcal{L}_{seg}$	IXI	MB42	Dice_small	42	0.0021	0.0020	ns	ns
$\mathcal{L}_{twce+seg}$ vs $\mathcal{L}_{seg}$	IXI	MB42	NSD_small	42	-0.0080	-0.0046	*	*
$\mathcal{L}_{twce+seg}$ vs $\mathcal{L}_{seg}$	IXI	AOMIC	Dice	46	-0.0022	-0.0025	*	*
$\mathcal{L}_{twce+seg}$ vs $\mathcal{L}_{seg}$	IXI	AOMIC	NSD	46	-0.0017	-0.0017	*	*
$\mathcal{L}_{twce+seg}$ vs $\mathcal{L}_{seg}$	IXI	AOMIC	Dice_small	46	0.0010	0.0023	ns	ns
$\mathcal{L}_{twce+seg}$ vs $\mathcal{L}_{seg}$	IXI	AOMIC	NSD_small	46	-0.0033	-0.0027	*	ns
$\mathcal{L}_{twce+seg}$ vs $\mathcal{L}_{seg}$	IXI	IXI	Dice	117	-0.0015	-0.0017	*	*
$\mathcal{L}_{twce+seg}$ vs $\mathcal{L}_{seg}$	IXI	IXI	NSD	117	-0.0011	-0.0012	*	*
$\mathcal{L}_{twce+seg}$ vs $\mathcal{L}_{seg}$	IXI	IXI	Dice_small	117	-0.0012	-0.0002	ns	ns
$\mathcal{L}_{twce+seg}$ vs $\mathcal{L}_{seg}$	IXI	IXI	NSD_small	117	-0.0028	-0.0025	*	*

Supplementary Figures

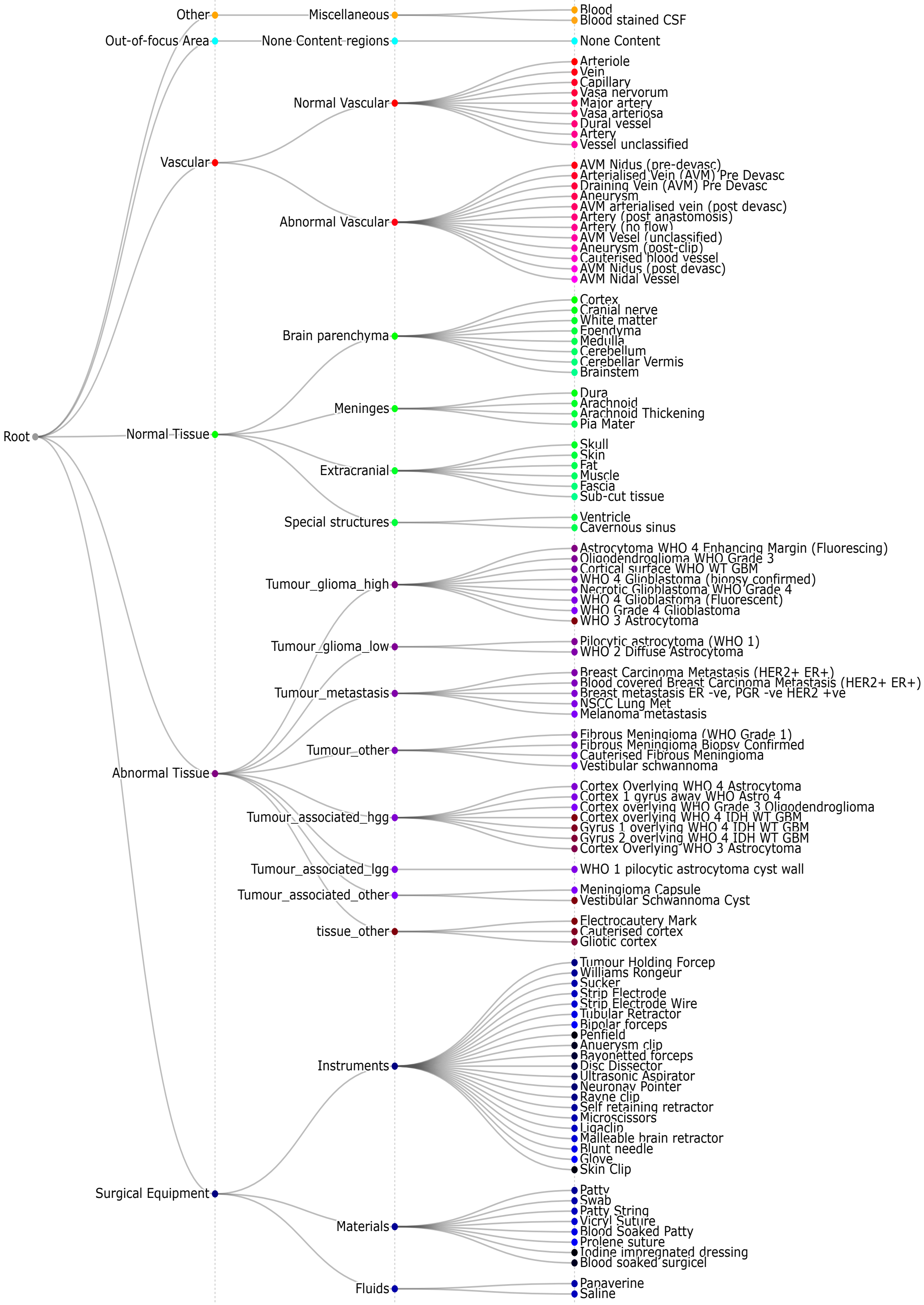

Figure S1: Full tree-based label hierarchy of the surgical HSI dataset. From left to right, the hierarchy progresses from coarse object categories to specific classes. The colour coding matches the ground-truth mask at each level.
